# Outcomes associated with antidepressant treatment according to the number of prescriptions and treatment changes: 5-year follow-up of a nation-wide cohort study

**DOI:** 10.3389/fpsyt.2022.923916

**Published:** 2022-09-08

**Authors:** Charles Ouazana-Vedrines, Thomas Lesuffleur, Anne Cuerq, Anne Fagot-Campagna, Antoine Rachas, Chrystelle Gastaldi-Ménager, Nicolas Hoertel, Frédéric Limosin, Cédric Lemogne, Philippe Tuppin

**Affiliations:** ^1^UFR de Médecine, Faculté de Santé, Université Paris Cité, Paris, France; ^2^Service de Psychiatrie de l’Adulte, AP-HP, Hôpital Hôtel-Dieu, Paris, France; ^3^Department of Pathologies and Patients, Caisse Nationale d’Assurance Maladie, Paris, France; ^4^INSERM U1266, Institut de Psychiatrie et Neurosciences de Paris, Université Paris Cité, Paris, France; ^5^Service de Psychiatrie et d’Addictologie de l’Adulte et du Sujet Âgé, AP-HP, Hôpital Corentin-Celton, Issy-les-Moulineaux, France

**Keywords:** administrative claims database, antidepressive agents, cohort studies, sick leaves, suicide

## Abstract

**Background:**

Naturalistic studies regarding clinical outcomes associated with antidepressant treatment duration have yielded conflicting results, possibly because they did not consider the occurrence of treatment changes. This nation-wide population-based study examined the association between the number of filled prescriptions and treatment changes and long-term psychiatric outcomes after antidepressant treatment initiation.

**Methods:**

Based on the French national health insurance database, 842,175 adults who initiated an antidepressant treatment in 2011 were included. Cox proportional-hazard multi-adjusted regression models examined the association between the number of filled prescriptions and the occurrence of treatment changes 12 months after initiation and four outcomes during a 5-year follow-up: psychiatric hospitalizations, suicide attempts, sick leaves for a psychiatric diagnosis, new episodes of antidepressant treatment.

**Results:**

During a mean follow-up of 4.5 years, the incidence rates of the four above-mentioned outcomes were 13.49, 2.47, 4.57, and 92.76 per 1,000 person-years, respectively. The number of filled prescriptions was associated with each outcome (adjusted HRs [95% CI] for one additional prescription ranging from 1.01 [1.00–1.02] to 1.10 [1.09–1.11]), as was the occurrence of at least one treatment change vs. none (adjusted HRs [95% CI] ranging from 1.18 [1.16–1.21] to 1.57 [1.79–1.65]). Furthermore, the adjusted HRs [95% CI] of the number of filled prescriptions were greater in patients with (vs. without) a treatment change for psychiatric hospitalizations (1.12 [1.11–1.14] vs. 1.09 [1.08–1.10], *p* for interaction = 0.002) and suicide attempts (1.12 [1.09–1.15] vs. 1.06 [1.04–1.08], *p* for interaction = 0.006).

**Limitations:**

Lack of clinical data about the disorders warranting the prescriptions or their severity.

**Conclusion:**

Considering treatment changes is critical when using administrative claims database to examine the long-term psychiatric outcomes of antidepressant treatments in real-life settings.

## Introduction

Depressive and anxiety disorders are leading causes of disability worldwide and are associated with an increased risk of suicide (1–3). Among pharmacologic interventions, antidepressant medications are the first-line treatment for unipolar major depressive episodes with moderate to severe intensity as well as for most anxiety disorders, apart from a specific phobia (4–6). Regarding major depression, about 50% of patients respond to a first-line antidepressant drug (i.e., a reduction of at least 50% of the intensity of depressive symptoms) but only 30% achieve full symptom remission (7). For those who remit from major depression, randomized controlled trials (RCTs) showed that discontinuation of antidepressant treatment in the first 6 months is associated with an increased risk of relapse (8). Therefore, guidelines generally recommend continuing the same medication at the same dosage for at least six months (4–6). However, participants in RCTs for depressive or anxiety disorders are not representative of patients encountered in real-life settings (9–12). Furthermore, 40% of patients at best complete a minimum 6-month duration of treatment in naturalistic studies (13–16). Real-life studies are thus needed to appreciate the consequences of these shorter durations of treatment.

Results from naturalistic studies regarding the short-term clinical correlates of a short duration of antidepressant treatment have been conflicting so far. Some studies have found that early discontinuation is associated with greater rates of poor psychiatric outcomes, including new antidepressant treatment, suicide attempts, mental health-related emergency department visits and psychiatric hospitalization (17, 18). Patients with stable use may be those at lower risk of relapse or recurrence (19). In contrast, other studies based on Dutch and French national claim databases found that early discontinuation was associated with a lower risk of reinitiating antidepressant treatment compared to persistent users (16, 20). One reason for these discrepancies is that a treatment duration of at least 6 months might be observed in two populations facing opposite situations: on one hand, individuals who have experienced a response and acceptable tolerance and who continue the same antidepressant treatment; on the other hand, those who experienced poor tolerance or no response and who were prescribed another treatment. Better outcomes are expected for the former than the later population, but studies that merged the two might have blurred these differences (16, 20).

The present study was based on the French national health insurance database (*Système National des Données de Santé*, SNDS), taking advantage of a prior study that undertook the follow-up of nearly one million patients who initiated an antidepressant treatment in 2011 (13). The present study had two aims: (i) describing long-term psychiatric outcomes (i.e., psychiatric hospitalization, suicide attempt, sick leave or sickness invalidity, new episode of antidepressant treatment) following the first prescription of an antidepressant, and (ii) determining whether these outcomes are associated with the number of filled prescriptions and the occurrence of a treatment change (i.e., either another drug or a combination of drugs) over a 12-month period. We hypothesized that a higher number of filled prescriptions would be associated with worse outcomes only in participants for whom different drugs were prescribed, suggesting poor efficacy, poor tolerability, or both.

## Materials and methods

### Data source

The SNDS collects the individual characteristics of the beneficiaries of the various French national health insurance schemes, as well as prescriptions and procedures performed on an outpatient basis and funded or reimbursed by the national health insurance (21). Individual characteristics include age, gender, commune of residence, vital status (date of death). A social deprivation index is also available at the scale of the commune, which is the smallest French administrative unit, based on household income, education level, occupational grade, and unemployment rate (22). The higher the index, the higher the level of social deprivation. The index could not be calculated for French overseas territories that were thus identified specifically. Patients from overseas territories were considered in the analysis as belonging to a specific category of this index.

Reimbursed drugs are identified according to the Anatomical Therapeutic Classification (ATC). The drugs dispensed during hospitalization are not reimbursed individually and cannot thus be identified. The SNDS does not record any clinical information related to consultations, prescriptions, or examinations. However, it includes information on diagnoses of long-term diseases eligible for 100% reimbursement of health care, including mental disorders as major depression. The SNDS also contains all admissions in public and private hospitals, including psychiatric hospitals, with diagnoses at discharge. Clinical diagnoses are also available for patients with sick leave for more than 180 days or disability allowance. All diagnoses are coded according to the International Classification of Diseases, 10th revision (ICD-10).

Forty-seven non-exclusive groups of diseases are identified in the SNDS, based on algorithm accounting for principal diagnoses, related or significant associated diagnoses in short-stay hospitals and psychiatric hospitals, long-term diseases, dispensing of specific drugs, and specific procedures (23). People with chronic diseases or treatments could thus be identified.

### Study population

This study was based on data from the general and local mutualist sections health insurance schemes covering 86% of the 66 million inhabitants due to the lack of completeness of certain data in the other schemes during the study period.

People were included at the date of their first reimbursement of an antidepressant from 1 January 2011 to 31 December 2011. They were identified by ATC codes starting with “N06A.” In France, there is no insurance restriction regarding the antidepressant medications used in this analysis. For amitriptyline (ATC code N06AA09), filled prescriptions of less than 1500 mg per prescription (i.e., presumably less than 50 mg per day, under the assumption of a prescription for at least one month) were excluded from our analyses, since low dosage amitriptyline is frequently used as an analgesic rather than an antidepressant agent (24).

**FIGURE 1:**
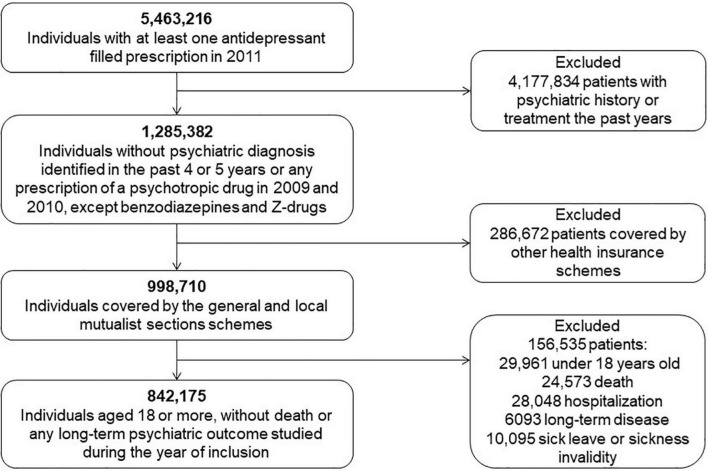
Flow chart of the study population selection.

In 2011, nearly 5.5 million people from the population source had at least one antidepressant filled prescription and among them, 1.3 million were defined as new antidepressant users in 2011 (Figure 1). New users were defined by the absence of any prescription of a psychotropic drug in 2009 and 2010, except benzodiazepines (N05BA) and Z-drugs (N05CF01, N05CF02), together with no prior psychiatric diagnosis (i.e., an ICD-10 F code, excluding codes F00–F03) identified in the past 4 or 5 years from long-term diseases or diagnoses at discharge after a hospitalization.

Then about one million participants were selected (Figure 1). Next, we excluded people aged less than 18 as well as those who died or experienced at least one of the long-term psychiatric outcomes described below during the 12 months after the first filled prescription, excepted for the resumption of antidepressant treatment after 12 months of abstinence. Finally, this study included 842,175 participants (Table 1).

**TABLE 1:** Characteristics of the population according to the number of filled antidepressant prescriptions.

		Filled prescriptions			
	Total	1	2	3–5	≥6
*N*	842,175	348,457	115,506	143,829	234,383
Row %	100.0	41.4	13.7	17.1	27.8
	%	%	%	%	%
**Female gender**	67.1	66.6	66.3	66.7	68.5
**Age (years)**					
<30	13.2	16.6	14.8	12.6	8.2
30–39	18.7	19.9	19.7	19.5	16.4
40–49	21.5	21.0	21.7	22.8	21.6
50–59	18.1	17.3	18.0	18.6	19.4
60–69	11.2	11.0	10.8	10.9	11.6
≥70	17.2	14.2	15.0	15.6	22.9
**Deprivation index (quintiles)**					
1 less deprived	18.0	17.0	17.3	18.3	19.5
2	19.0	18.5	18.4	19.2	19.8
3	19.9	19.7	19.6	19.9	20.3
4	19.9	19.9	20.1	19.9	19.8
5 more deprived	21.3	22.4	22.5	20.9	19.5
Overseas territories	1.9	2.5	2.1	1.7	1.0
Chronic disease*					
At least one neurodegenerative disease or dementia	3.2	1.8	2.2	2.6	3.6
At least another chronic non-psychiatric disease	19.8	18.2	19.0	19.2	22.8
**Season**					
January–March	28.5	28.3	28.6	29.0	28.4
April–June	25.6	25.5	25.8	25.8	25.5
July–September	22.5	22.6	22.4	21.9	22.9
October–December	23.4	23.5	23.2	23.3	23.2
**Psychiatrist potential localized accessibility (quartiles)**					
1 less accessible	24.2	24.7	24.7	23.9	23.4
2	26.0	26.1	26.1	25.8	26.0
3	25.1	24.9	24.8	25.4	25.5
4 more accessible	24.7	24.3	24.4	24.9	25.1
**First prescriber**					
GP or hospital practitioner	89.2	90.4	89.5	88.7	87.2
Psychiatrist, private practice	5.3	3.7	4.7	5.9	8.1
Another specialist, private practice	5.5	5.9	5.8	5.4	4.8
Psychotropic drugs reimbursed**					
Mood stabilizers	0.9	0.5	0.8	1.0	1.4
Atypical antipsychotics	1.6	0.7	1.1	1.7	3.0
Typical antipsychotics	2.2	1.2	2.0	2.5	3.6
Z-drugs	22.8	17.7	23.3	25.8	28.4
Benzodiazepines	52.0	43.8	53.1	57.1	60.7
**At least one antidepressant change**			23.7	28.1	23.9

GP, General Practitioner; SD, Standard Deviation.

*In the SNDS, algorithms identify 47 non-exclusive groups of chronic non-psychiatric diseases.

**At least one filled prescription in the year of inclusion.

All between-category comparisons were significant with a p-value < 0.001.

### Number of filled prescriptions and treatment changes

After the first filled prescription between 1 January 2011 and 31 December 2011, we considered both the number of filled prescriptions and the number of treatment changes during the following 12 months (i.e., up to 31 December 2012). Most guidelines recommend a prescription duration of at least 6 months in case of efficacy and tolerance. However, since we aimed at being able to document treatment changes that may occur after more than one filled prescription of the initial treatment, we consider a 12-month follow-up, also allowing for these changes to occur later in clinical practice than as recommended in guidelines. The number of filled antidepressant prescriptions that qualitatively differed from the first one (i.e., either a different antidepressant or a combination of antidepressants not prescribed so far) was used to estimate the number of treatment changes. For instance, after a first prescription of the antidepressant A, a filled prescription of another antidepressant B either alone or in combination with A counted as one change. Should a filled prescription of an antidepressant C, either alone or in combination with A or B, be later observed, two changes were considered and so on.

### Long-term psychiatric outcomes

The follow-up period started from 12 months after the first filled prescription to 31 December 2016. The following outcomes were considered: (i) hospitalization in a psychiatric hospital; (ii) hospitalization in a general hospital after a suicide attempt (i.e., ICD-10 codes T36–T50 or X60–X84); (iii) sick leave > 180 days or sickness invalidity for mental disorders; (iv) new episode of antidepressant treatment (i.e., the presence of at least one filled prescription at any time during the follow-up period when there was no filled prescription in the previous 12 months). Mental disorders were considered using an ICD-10 F code, excluding codes F00–F03.

For sick leave > 180 days or sickness invalidity for mental disorders, the population study was restricted to people aged less than 60, because these invalidities are recorded in a different way after 60 years in the SNDS.

### Covariates

In addition to gender and age, the covariates included the social deprivation index (see above) and the following variables.

Participants with chronic non-psychiatric diseases were identified based on the 47 groups of non-psychiatric diseases or non-psychotropic drugs of the above-mentioned tool. Two binary variables were considered: at least one neurodegenerative disease (including Parkinson’s disease) or dementia, at least another chronic non-psychiatric disease.

The season of the first filled prescription was categorized as a four-class variable: January–March, April–June, July–September, October–December, to account for a potential effect of the length of washout period as well as for any seasonal effect (e.g., seasonal mood variations, availability of physicians).

The commune of residence was used to determine the potential localized accessibility of health care delivered by psychiatrists (private practice). This indicator takes into account, on the one hand, practitioners’ volume of activity in the commune and in the neighboring commune and, on the other hand, the rates of use of services differentiated according to the age structure of the population in each French commune. This indicator also takes into account a function of services use balanced with the remoteness of the population to health care supply. Better accessibility is designated by a higher indicator (25).

The specialty of the private physician who prescribed the first antidepressant was collected from the SNDS database and classified into three categories: general practitioners (GP) or hospital practitioner; psychiatrist, private practice; another specialist, private practice.

Treatments by mood stabilizers, atypical antipsychotics, typical antipsychotics, Z-drugs and benzodiazepines during the 12 months after the first filled prescription were considered as binary variables (at least one filled prescription vs. none).

### Statistical analyses

In the descriptive analyses, patients were classified into four subgroups according to the number of filled prescriptions during the 12 months after the first prescription (one, two, three to five, six or more filled prescriptions). The choice of these categories was *a priori* driven by several reasons. First, since only one filled prescription without subsequent refill may be of uncertain meaning regarding treatment indication or medication adherence (26), the reference category for the analyses related to the number of filled prescriptions was two filled prescriptions. Second, the choice of the highest category was based on the above-mentioned guidelines that generally recommend continuing the same medication at the same dosage for at least 6 months. Finally, since we had no *a priori* reason to hypothesize a linear relationship, we did not distinguish between those with three to five filled prescriptions. In addition, patients were classified into three subgroups according to the number of treatment changes during the 12 months after the first prescription (none, one, two or more treatment changes). These subgroups were compared with Chi-square tests for categorical variables, described as percentages. The incidence rate (IR) of each long-term psychiatric outcome (i.e., psychiatric hospitalization; suicide attempt; sick leave > 180 days or sickness invalidity for mental disorders among those < 60 years; new episode of treatment) from 12 months after the first filled prescription to 31 December 2016 was computed as the number of events per 1000 person-years.

In the main analyses, Cox proportional-hazard regression models were used to compute hazard ratios (HR) and 95% confidence interval (CI) for each long-term outcome, adjusting for the following variables at inclusion: gender, age, social deprivation index, presence of chronic non-psychiatric disease, season, psychiatrist potential localized accessibility, first prescriber’s specialty, other psychotropic drug refills. Proportional hazards assumptions were tested by adding an interaction between time and other variables. First, the number of filled prescriptions during the 12 months after the first prescription was used as a categorical variable (one, two, three to five, six or more filled prescriptions). Then, to test whether the associations between the number of filled prescriptions and the long-term psychiatric outcomes depended upon the occurrence of treatment changes, additional adjusted regression models were computed with both the number of filled prescriptions (as a continuous variable) and the occurrence of at least one treatment change (at least one vs. none) among patients with at least two filled prescriptions. This was done to allow searching for an interaction between the occurrence of at least one treatment change (vs. none) and the number of filled prescriptions. The number of filled prescriptions was used as a continuous variable in these analyses, based on the apparent gradient observed with the categorical variable and the four outcomes in the descriptive analyses (see section “Results”) whereas the occurrence of treatment change was used as a binary variable to reduce the risk of collinearity between the two variables. Should this interaction be significant, adjusted HRs and 95% CI for the number of filled prescriptions were computed separately for the patients with and without at least one treatment change.

Statistical analyses were performed with SAS 9.3 software (SAS Institute Inc., Cary, NC, United States) with a threshold for statistical significance at two-sided *p* < 0.05. The CNAM, as a health research institute, has permanent access to the SNDS database approved by decree and the French data protection authority (*Commission Nationale de l’Informatique et des Libertés).*

## Results

### Characteristics of the study population

The characteristics of the included individuals are displayed in Table 1. Their mean age was 50.1 years (standard deviation: 18.1), 67.1% were females, the first prescriber was mainly a GP or a hospital practitioner (89.2%), 3.2% had a neurodegenerative disease or dementia and 19.8% at least another chronic non-psychiatric disease, and 0.9, 1.6, 2.2, 22.8, and 52.0% had at least one filled prescription of a mood-stabilizer, atypical antipsychotic drug, typical antipsychotic drug, Z-drug and benzodiazepine, respectively. During the 12 months after the first filled prescription, 41% had only one filled antidepressant prescription, 14% two, 17% three to five, and 28% six or more. All between-category comparisons were significant with a *p*-value < 0.001.

### Incidence of long-term psychiatric outcomes

During a mean follow-up of 4.5 years, 50,992 participants (incidence rate: 13.49 per 1,000 person-years) had at least one psychiatric hospitalization, 9584 (incidence rate: 2.47 per 1,000 person-years) had at least one least one hospitalization for a suicide attempt, 12,967 (incidence rate: 4.57 per 1,000 person-years) had a sick leave > 6 months or sickness invalidity for a psychiatric disease (<60 years) and 204,672 (incidence rate: 92.76 per 1,000 person-years) had a new episode of antidepressant treatment. Among a total of 3,745,018 person-years, the outcome rates and the IR/1,000 person-years increased with a gradient from one filled prescription to six or more and from no treatment change to two or more treatment changes (Table 2).

**TABLE 2:** Crude incident rate for each long-term psychiatric outcome according to the number of filled antidepressant prescriptions and treatment changes.

	Filled prescriptions				Treatment changes		
	1	2	3-5	≥ 6	0	1	≥ 2
N	348,457	115,506	143,829	234,383	178,428	33,735	22,220
**At least one psychiatric hospitalization**							
%	3.89	5.21	6.07	8.50	7.12	10.50	14.91
PY	1,524,812	511,239	649,210	1,093,635	822,230	165,467	119,611
IR/1000 PY	9.02	12.11	14.11	20.04	16.73	25.36	37.27
**At least one hospitalization for a suicide attempt**							
%	0.76	1.07	1.18	1.50	1.16	1.95	3.09
PY	1,551,554	523,551	668,463	1,142,598	844,333	172,826	128,012
IR/1000 PY	1.73	2,42	2,66	3.38	2.63	4.41	6.95
**Sick leave >6 months or sickness invalidity for a psychiatric diagnosis (<60 years old)**							
N	260,697	85,728	105,694	150,634	112,862	22,406	15,366
%	1.49	1.99	2.29	2.80	3.38	5.29	8.45
PY	1,162,041	391,843	499,968	782,222	836,681	170,434	124,812
IR/1000 PY	3.37	4.49	5.10	6.02	7.73	12.26	19.90
**New episode of antidepressant treatment after 12 months of abstinence**							
N	324,567	106,112	126,891	153,296	116,395	22,389	14,512
%	21.45	27.10	30.34	37.71	35.82	40.67	45.86
PY	1,068,074	334,406	389,090	414,963	309,769	61,969	43,225
IR/1000 PY	66.01	88.36	103.76	154.82	144.30	172.04	205.53

CI: Confidence Interval, HR: Hazard Ratio, IR: Incidence Rate, PY: person-year.

### Associations of long-term psychiatric outcomes with the number of filled prescriptions

In multi-adjusted models, the number of filled prescriptions as a categorical variable was positively associated with each outcome with adjusted HR (95% CI) for at least six filled prescriptions vs. only two ranging from 1.05 (1.04–1.07) for a new episode of antidepressant treatment after 12 months of abstinence to 1.38 (1.34–1.42) for at least one psychiatric hospitalization (Table 3). Consistent with the gradient observed in unadjusted analysis (Table 2), intermediate adjusted HR (95% CI) were observed for 3–5 filled prescriptions.

**TABLE 3:** Hazard ratios for each long-term psychiatric outcome according to the number of filled antidepressant prescriptions.

Outcomes	At least one psychiatric hospitalization		At least one hospitalization for a suicide attempt		Sick leave > 6 months or sickness invalidity for a psychiatric disease (<60 years)		New episode of antidepressant treatment	
	HR	95% CI	HR	95% CI	HR	95% CI	HR	95% CI
**Filled prescriptions**								
1	0.80	0.78–0.83	0.79	0.74–0.85	0.81	0.76–0.86	0.81	0.80–0.82
2	Ref.		Ref.		Ref.		Ref.	
3–5	1.10	1.06-1.13	1.02	0.94–1.10	1.10	1.03–1.17	1.02	1.00–1.05
≥6	1.38	1.34–1.42	1.26	1.17–1.34	1.27	1.19–1.34	1.05	1.04–1.07
**Female gender**	0.75	0.73–0.76	0.98	0.94–1.03	1.08	1.04–1.13	1.18	1.17–1.20
**Age** years								
<30	Ref.		Ref.		Ref.		Ref.	
30–40	0.83	0.81–0.86	0.78	0.73–0.83	1.44	1.36–1.53	1.18	1.16–1.20
40–50	0.78	0.76–0.81	0.70	0.66–0.74	1.41	1.33–1.50	1.24	1.22–1.26
50–60	0.60	0.57–0.62	0.42	0.39–0.45	1.27	1.19–1.35	1.13	1.10–1.15
60–70	0.58	0.55–0.60	0.24	0.22–0.26			1.08	1.04–1.11
≥70	1.16	1.12–1.19	0.24	0.21–0.26			1.12	1.10–1.14
**Deprivation index (quintiles)**								
1 less deprived	Ref.		Ref.		Ref.		Ref.	
2	1.05	1.02–1.08	1.18	1.09–1.27	1.01	0.96–1.08	1.00	0.98–1.01
3	1.10	1.071.13	1.25	1.16–1.34	1.02	0.96–1.08	0.99	0.97–1.00
4	1.13	1.10–1.17	1.34	1/25–1.45	0.96	0.91–1.03	0.99	0.98–1.01
5 more deprived	1.19	1.15–1.22	1.46	1.36–1.57	0.86	0.81–0.92	1.01	0.99–1.02
Overseas territories	1.36	1.27–1.46	0.90	0.74–1.10	0.51	0.41–0.63	0.84	0.81–0.87
**Chronic disease^a^**								
At least one neurodegenerative disease or dementia	1.30	1.24–1.35	1.01	0.86–1.17	0.34	0.26–0.46	0.86	0.83–0.88
At least another chronic non-psychiatric disease	1.12	1.09–1.15	1.05	0.99–1.12	0.87	0.82–0.92	1.01	0.99–1.02
**Season**								
January–March	Ref.		Ref.		Ref.		Ref.	
April–June	1.02	0.99–1.04	0.99	0.93–1.04	0.95	0.91–0.99	1.01	1.00–1.02
July–September	1.04	1.01–1.06	1.03	0.97–1.09	0.87	0.83–0.92	0.99	0.98–1.01
October–December	0.99	0.97–1.02	0.98	0.93–1.04	0.83	0.78–0.87	0.99	0.98–1.00
**Psychiatrist potential localized accessibility (quartiles)**								
1 less accessible	Ref.		Ref.		Ref.		Ref.	
2	0.99	0.96–1.01	0.97	0.92–1.03	1.03	0.97–1.09	1.01	1.00–1.02
3	0.97	0.94–0.99	0.88	0.83–0.94	1.12	1.06–1.19	1.02	1.01–1.03
4 more accessible	0.99	0.96–1.02	0.74	0.70–0.80	1.18	1.11–1.25	1.05	1.04–1.07
**First prescriber**								
GP or hospital practitioner	Ref.		Ref.		Ref.		Ref.	
Psychiatrist, private practice	1.03	0.99–1.07	0.89	0.82–0.97	1.08	1.00–1.14	0.98	0.96–1.00
Another specialist, private practice	0.93	0.89–0.97	0.86	0.76–0.96	0.75	0.68–0.83	0.98	0.96–1.00
**Psychotropic drugs reimbursed^b^**								
Mood stabilizers	1.74	1.65–1.83	1.66	1.48–1.85	1.21	1.05–1.39	1.16	1.12–1.21
Atypical antipsychotics	2.41	2.33–2.50	1.75	1.60–1.92	1.08	0.97–1.21	1.17	1.13–1.20
Typical antipsychotics	2.00	1.93–2.07	2.28	2.11–2.46	1.10	1.00–1.21	1.19	1.16–1.22
Z-drugs	1.39	1.36–1.42	1.78	1.71–1.86	1.27	1.22–1.33	1.11	1.10–1.12
Benzodiazepines	1.55	1.52–1.58	1.90	1.82–2.00	1.32	1.27–1.38	1.17	1.15–1.18

CI, Confidence Interval; HR, Multivariable Adjusted Hazard Ratio.

^a^In the SNDS, algorithms identify 47 non-exclusive groups of chronic non-psychiatric diseases.

^b^At least one filled prescription in the year of inclusion.

### Associations of long-term psychiatric outcomes with the number of filled prescriptions and the occurrence of at least one treatment change among patients with a least two filled prescriptions

In multi-adjusted models, the number of filled prescriptions as a continuous variable was positively associated with each outcome with adjusted HR (95% CI) for each additional prescription ranging from 1.01 (1.00–1.04) for a new episode of antidepressant treatment after 12 months of abstinence to 1.10 (1.09–1.11) for at least one psychiatric hospitalization (Table 4). Meanwhile, the occurrence of at least one treatment change was positively associated with each outcome with adjusted HR (95% CI) ranging from 1.18 (1.16–1.21) for a new episode of antidepressant treatment after 12 months of abstinence to 1.57 (95% CI: 1.49–1.65) for at least one least one hospitalization for a suicide attempt (Table 4).

**TABLE 4:** Hazard ratios for each long-term psychiatric outcome according to the occurrence of at least one antidepressant change and the number of filled prescriptions among patients with a least two filled prescriptions.

Outcomes	At least one psychiatric hospitalization		At least one hospitalization for a suicide attempt		Sick leave > 6 months or sickness invalidity for a psychiatric disease (<60 years)		New episode of antidepressant treatment	
	HR	95% CI	HR	95% CI	HR	95% CI	HR	95% CI
**At least one antidepressant change (vs. none)**	1.49	1.46–1.53	1.57	1.49–1.65	1.38	1.32–1.45	1.18	1.16–1.21
**Number of filled prescriptions^a^**	1.10	1.09–1.11	1.08	1.06–1.10	1.07	1.05–1.08	1.01*	1.00–1.02
**Female gender**	0.76	0.74–0.78	1.02	0.97–1.08	1.03	0.98–1.08	1.13	1.12–1.15
**Age (years)**								
<30	Ref.		Ref.		Ref.		Ref.	
30–40	0.83	0.80–0.86	0.80	0.74–0.86	1.40	1.30–1.51	1.12	1.10–1.15
40–50	0.80	0.77–0.83	0.73	0.68–0.78	1.36	1.26–1.47	1.16	1.13–1.18
50–60	0.61	0.58–0.63	0.45	0.41–0.49	1.26	1.17–1.36	1.06	1.04–1.09
60–70	0.60	0.57–0.63	0.26	0.23–0.30			0.94	0.91–0.96
≥70	1.07	1.03–1.11	0.21	0.19–0.24			0.96	0.94–0.98
**Deprivation index (quintiles)**								
1 less deprived	Ref.		Ref.		Ref.		Ref.	
2	1.05	1.01–1.09	1.19	1.09–1.30	1.01	0.94–1.08	1.00	0.98–1.01
3	1.09	1.06–1.13	1.23	1.13–1.34	1.01	0.95–1.08	0.99	0.97–1.00
4	1.14	1.10–1.18	1.36	1.24–1.48	0.95	0.88–1.02	0.99	0.97–1.01
5 more deprived	1.18	1.14–1.22	1.43	1.31–1.56	0.86	0.79–0.92	1.00	0.98–1.02
Overseas territories	1.27	1.17–1.39	0.75	0.57–0.99	0.60	0.46–0.78	0.93	0.88–0.98
**Chronic disease^b^**								
At least one neurodegenerative disease or dementia	1.20	1.14–1.26	0.93	0.78–1.10	0.32	0.23–0.45	0.90	0.87–0.93
At least another chronic non-psychiatric disease	1.08	1.05–1.11	1.05	0.97–1.13	0.83	0.77–0.89	0.99	0.97–1.00
**Season**								
January–March	Ref.		Ref.		Ref.		Ref.	
April–June	1.00	0.98–1.03	1.00	0.94–1.07	0.94	0.89–1.00	1.01	0.99–1.02
July–September	1.02	0.99–1.05	1.02	0.95–1.09	0.87	0.81–0.92	1.00	0.98–1.01
October–December	0.97	0.95–1.00	0.95	0.89–1.02	0.84	0.79–0.90	0.98	0.97–1.00
**Psychiatrist potential localized accessibility (quartiles)**								
1 less accessible	Ref.		Ref.		Ref.		Ref.	
2	0.99	0.96–1.02	0.97	0.90–1.03	1.05	0.99–1.13	1.01	0.99–1.03
3	0.96	0.93–0.99	0.85	0.79–0.91	1.15	1.07–1.23	1.03	1.01–1.05
4 more accessible	0.97	0.94–1.00	0.74	0.69–0.80	1.17	1.10–1.26	1.05	1.03–1.07
**First prescriber**								
GP or hospital practitioner	Ref.		Ref.		Ref.		Ref.	
Psychiatrist, private practice	0.97	0.94–1.01	0.84	0.76–0.92	1.01	0.94–1.10	0.96	0.93–0.98
Another specialist, private practice	0.89	0.84–0.94	0.77	0.67–0.88	0.70	0.61–0.79	0.96	0.94–0.99
**Psychotropic drugs reimbursed^c^**								
Mood stabilizers	1.77	1.68–1.86	1.74	1.54–1.96	1.18	1.04–1.37	1.13	1.09–1.18
Atypical antipsychotics	2.42	2.33–2.52	1.86	1.69–2.05	1.08	0.96–1.22	1.20	1.16–1.23
Typical antipsychotics	2.05	1.97–2.12	2.46	2.27–2.67	1.14	1.04–1.26	1.18	1.15–1.21
Z-drugs	1.16	1.13–1.19	1.40	1.32–1.49	1.12	1.06–1.19	1.07	1.06–1.09
Benzodiazepines	1.21	1.18–1.24	1.24	1.18–1.31	1.17	1.12–1.23	1.10	1.08–1.11

CI, Confidence Interval; HR, Adjusted Hazard Ratio.

^a^The “number of filled prescriptions” was considered a continuous variable so the HR relates to an increment of 1 filled prescription.

^b^In the SNDS, algorithms identify 47 non-exclusive groups of chronic non-psychiatric diseases.

^c^At least one filled prescription in the year of inclusion.

*Statistically significant when the Confidence Interval includes 1.00.

When entering in the regression model the interaction between the occurrence of at least one antidepressant change and the number of filled prescriptions, this interaction was significant for both psychiatric hospitalizations (p = 0.002) and hospitalizations for suicide attempt (p = 0.006).

The interaction between the occurrence of at least one treatment change and the number of filled prescriptions was significant for both psychiatric hospitalizations (*p* = 0.002) and hospitalizations for suicide attempt (*p* = 0.006). The adjusted HRs for the number of filled prescriptions were significantly greater in patients with a least one treatment change than in those without for both psychiatric hospitalizations (1.12 vs. 1.09) and hospitalizations for suicide attempt (1.12 vs. 1.06) (Table 5).

**TABLE 5:** Hazard ratios for the two long-term psychiatric outcomes for which there was a significant interaction between the occurrence of at least one antidepressant change and the number of filled prescriptions among patients with a least two filled prescriptions.

Outcomes	At least one psychiatric hospitalization		At least one hospitalization for a suicide attempt	
	HR	95% CI	HR	95% CI
**Number of filled prescriptions (patients with at least one antidepressant change)^a^**	1.12	1.11–1.14	1.12	1.09–1.15
**Number of filled prescriptions (patients with no antidepressant change)^a^**	1.09	1.08–1.10	1.06	1.04–1.08
**Female gender**	0.76	0.74–0.78	1.02	0.97–1.08
**Age (years)**				
<30	Ref.		Ref.	
30–40	0.83	0.79–0.86	0.80	0.74–0.86
40–50	0.80	0.77–0.83	0.73	0.68–0.79
50–60	0.61	0.58–0.63	0.44	0.41–0.48
60–70	0.60	0.57–0.63	0.26	0.23–0.30
≥70	1.07	1.03–1.11	0.21	0.19–0.24
**Deprivation index (quintiles)**				
1 less deprived	Ref.		Ref.	
2	1.05	1.01–1.09	1.19	1.09–1.30
3	1.09	1.06–1.13	1.23	1.13–1.34
4	1.14	1.10–1.18	1.36	1.24–1.48
5 more deprived	1.18	1.14–1.22	1.43	1.31–1.56
Overseas territories	1.28	1.17–1.14	0.75	0.58–0.99
**Chronic disease^b^**				
At least one neurodegenerative disease or dementia	1.20	1.14–1.26	0.92	0.78–1.11
At least another chronic non-psychiatric disease	1.08	1.05–1.11	1.05	0.97–1.13
**Season**				
January–March	Ref.		Ref.	
April–June	1.05	0.98–1.03	0.99	0.94–1.07
July–September	1.03	1.00–1.06	1.00	0.95–1.09
October–December	0.98	0.95–1.00	0.95	0.90–1.03
**Psychiatrist potential localized accessibility (quartiles)**				
1 less accessible	Ref.		Ref.	
2	0.99	0.96–1.02	0.97	0.90–1.03
3	0.96	0.93–0.99	0.85	0.79–0.91
4 more accessible	0.97	0.94–1.00	0.74	0.69–0.80
**First prescriber**				
GP or hospital practitioner	Ref.		Ref.	
Psychiatrist, private practice	0.97	0.94–1.02	0.84	0.76–0.92
Another specialist, private practice	0.89	0.84–0.94	0.77	0.67–0.88
**Psychotropic drugs reimbursed^c^**				
Mood stabilizers	1.76	1.67–1.86	1.73	1.53–1.95
Atypical antipsychotics	2.41	2.31–2.49	1.85	1.68–2.04
Typical antipsychotics	2.04	1.97–2.12	2.45	2.26–2.66
Z-drugs	1.16	1.13–1.19	1.40	1.32–1.48
Benzodiazepines	1.21	1.18–1.23	1.24	1.18–1.31

CI, Confidence Interval; HR, Adjusted Hazard Ratio.

^a^The “number of filled prescriptions” was considered a continuous variable so the HR relates to an increment of 1 filled prescription.

^b^In the SNDS, algorithms identify 47 non-exclusive groups of chronic non-psychiatric diseases.

^c^At least one filled prescription in the year of inclusion.

## Discussion

### Summary of results

In a nationwide population of nearly one million of new users of antidepressants, the number of filled prescriptions and the occurrence of at least one treatment change in the 12 months following the first prescription were both associated with worse long-term psychiatric outcomes. Regarding the number of filled prescriptions, this association even suggested a dose-response relationship. Furthermore, for both psychiatric hospitalizations and hospitalizations for suicide attempts, the positive associations with the number of filled prescriptions were significantly greater in patients with at least one treatment change than in those without.

### Strengths and limitations

Strengths include the sample size, the duration of the follow-up period, the use of several outcomes, the generalizability of our results and their novelty. Specifically, to our knowledge, this study is the first to show that considering the number of treatment lines is critical when studying the psychiatric outcomes associated with the continuation vs. early discontinuation of antidepressants. Our results were consistent with previous population-based studies, notably regarding early cessation of antidepressant medications, which was reported to occur in about 45% of cases after a first prescription in the general population (27). However, taking as the reference category the patients with two filled prescriptions, our results were not blurred by outcomes observed among those with only one filled prescription. Although the presence of at least two filled prescriptions suggest that the patients did take the first one and that a prescribing physician confirmed the indication, both treatment indication and medication adherence might be more questionable for those with only one filled prescription. For instance, the proportion of individuals who did not actually need an antidepressant treatment is likely greater in patients with only one filled prescription than in those with at least two prescriptions. Furthermore, at least 12 months without filled prescriptions were required to consider a new episode of treatment, making our results regarding this outcome unlikely to be explained by withdrawal effects after treatment cessation. This later point is worth mentioning as a substantial proportion of antidepressant users who come off the medication experience withdrawal reactions that may be misdiagnosed as relapse (28).

Some limitations should be acknowledged. First, although the wealth of data collected in the SNDS allowed taking into account comorbidity, it did not generate information about the disorders that warranted the prescription of antidepressant medications or about their severity. For instance, antidepressant medications are also used to treat neuropathic pain. To limit this flaw, for amitriptyline, filled prescriptions of less than 1,500 mg per prescription (i.e., presumably less than 50 mg per day) were excluded from our analyses, since low dosage amitriptyline is frequently used as an analgesic agent. This method could not be used regarding other antidepressant medications that can be used to treat neuropathic pain (mainly duloxetine in France) as there is no difference in prescription dose range that may differentiate their use as antidepressant or analgesic agents. Furthermore, the psychiatric diagnoses warranting the hospitalization in psychiatric hospitals or data about psychotherapy were not available either. Second, the SNDS also does not provide data about actual medication consumption, prescriptions that were not filled by the patients, or medications that were delivered during hospitalizations. This last point probably did not affect our independent variables much since individuals with a prior psychiatric hospitalization or a psychiatric diagnosis at discharge after a hospitalization were not included in the present study. Third, we cannot exclude misclassification regarding chronic non-psychiatric diseases identified through algorithms from the SNDS. Fourth, the exclusion of patients who experienced one of the psychiatric outcomes during the first 12 months after treatment initiation may affect our results to some extent. However, it is noteworthy that we did find longer treatments being associated with worse outcomes, suggesting that this potential selection bias did not mask these associations as in other studies (20). Nevertheless, this bias may have led us to underestimate the magnitude of these association between longer treatments and worse outcome. Fifth, although treatment changes encompassed both adding a new antidepressant leading to a combination of antidepressants (i.e., antidepressants with the same date of reimbursement for a given patient) and switch (i.e., the reimbursement of another antidepressant without further new reimbursement of the first), we were not able to reliably distinguish between these two strategies. Sixth, data about suicide attempts may have high specificity but low sensitivity since suicide attempts were only recorded when they were followed by hospitalization in a general hospital. Seventh, the exclusion of individuals with at least one filled prescription for a mood-stabilizing medication reduced the risk of including patients with bipolar disorder but probably resulted in excluding some patients with unipolar depression. Eighth, the length of the wash-out period at inclusion was not equivalent for all participants as it was longer for those included at the end of 2011. However, adjusting for the season of inclusion allowed us to account for a potential effect of the length of washout period as well as for any seasonal effect (e.g., seasonal mood variations, availability of physicians). Ninth, excluding agricultural workers scheme and the self-employed workers scheme from the analysis due to lack of data might have resulted in a selection bias preventing generalization of our result to all the French population. However, these schemes provide the same access to mental health care as the general scheme. Tenth, we acknowledge that we could have also use treatment prescription duration as a determinant. However, when analyzing claim databases, the number of filled prescription might be a more meaningfull variable than treatment duration only (e.g., 6 months of treatment with only two filled prescriptions vs. six filled prescriptions of the same treatment). Also, since we tested two main predictive variables over four dependent variables, we cannot rule out that multiple testing may account for some significant results because of type I error. However, our results were consistent across the four dependent variables suggesting that significant results were unlikely to be chance findings. Finally, our study was research-oriented and did not aim to directly inform clinical practice. However, refining our knowledge about the long-term psychiatric outcomes following the first prescription of an antidepressant might be useful for clinicians. In addition, the previously shown association between a higher number of filled prescriptions and worse clinical outcomes might be interpreted as suggesting that long-term use of antidepressants could be detrimental. Accounting for the occurrence of treatment changes, our study might contribute to give a more balanced picture on this topic.

### Interpretation of findings

The association of a higher number of filled prescriptions with poorer long-term psychiatric outcomes is common in observational studies (16, 20, 29). This association is likely to be explained to some extent by the severity of the underlying condition, resistance to medications or indication bias (30). Individual factors such as a greater tendency to seek medical help as well as societal factors such as better access to health care may also partially account for this association beyond clinical features. Some of these poor outcomes may also be explained by detrimental neurophysiological adaptations to prolonged drug exposure or withdrawal effects (28). While our study was not designed to disentangle these hypotheses, we postulated that treatment changes, signaling poor efficacy, poor tolerability, or both, would be worth considering to better understand these associations. As mentioned in the introduction, considering only the number of filled prescriptions when examining the long-term psychiatric outcomes of a first antidepressant treatment might be misleading since it merges two populations facing two opposite situations: on the one hand, individuals who continue the same antidepressant treatment after having experienced a significant clinical improvement with acceptable tolerance; on the other hand, those to whom another treatment had been prescribed due to poor tolerance or low efficacy. The number of filled prescriptions and the occurrence of a treatment change were both positively associated with poorer clinical outcomes. Regarding the number of filled prescriptions, however, the apparent dose-response relationship was moderated by the occurrence of treatment changes for two outcomes. Specifically, psychiatric hospitalizations and hospitalizations for suicide attempts were associated with the number of filled prescriptions with a greater magnitude in patients with at least one treatment change than in those without. While not fully supporting our *a priori* hypothesis, the present results demonstrate that the occurrence of a treatment change may be associated with worse outcomes independent of the number of filled prescription. Furthermore, the occurrence of treatment changes may moderate the association between the number of filled prescriptions and, arguably, the two most important outcomes. Overall, these results support the need to scrutinize the number of treatment changes before interpreting the outcomes associated with the number of filled prescription of antidepressants.

Beside the correlates of the number of filled prescriptions or treatment changes, other results deserve to be highlighted. First, among a population of arguably first users of antidepressants, women were less likely than men to be hospitalized for psychiatric reasons, but more likely to experience a new episode of antidepressant treatment. This finding may be explained by a higher vulnerability for depression in women, while men might have to present with more severe symptoms to be diagnosed and treated with depression (31). Regarding age, our results indicate that the risk of being hospitalized for psychiatric reasons slowly goes down from individuals aged less than 30 to those aged 60–70 before dramatically rising in those aged 70 or more. This result is in line with the epidemiology of depressive and anxiety disorders in the general population, as is the decreased risk of hospitalization for non-fatal suicide attempts with increased age (32, 33). These findings are thus in line with the literature and provide further external validity to the present study.

### Prospects and conclusion

The present study provides evidence that the number of different treatment lines should be carefully considered when interpreting the clinical outcomes associated with the number of filled prescriptions of antidepressants. This finding is noteworthy in the context of using big data from administrative claims database to examine the effectiveness of antidepressants in real-life settings. For instance, although the raw number of filled prescriptions is unable to provide meaningful data about the clinical effectiveness of the related treatment, characterizing the sequence of these treatments may be a promising avenue to a more fruitful use of these data (26). Such approaches hold great promises in supplementing data from RCTs.

## Data availability statement

The original contributions presented in this study are included in the article/supplementary material, further inquiries can be directed to the corresponding author.

## Author contributions

CO-V, TL, AC, AF-C, CG-M, CL, and PT designed the study. TL and AC analyzed the data. CO-V, TL, CL, and PT wrote the first draft. All authors contributed to the interpretation of the data, revised the first draft critically for important intellectual content, finally approved of the version to be published, and agreed to be accountable for all aspects of the work.
